# Factorial validation of the patient assessment of chronic illness care (PACIC) and PACIC short version (PACIC-S) among cardiovascular disease patients in the Netherlands

**DOI:** 10.1186/1477-7525-10-104

**Published:** 2012-08-31

**Authors:** Jane Murray Cramm, Anna Petra Nieboer

**Affiliations:** 1 Institute of Health Policy & Management (iBMG), Erasmus University, Rotterdam, The Netherlands

**Keywords:** Disease management, Chronic care model, Chronic illness care, Quality, Primary care, Cardiovascular diseases

## Abstract

**Objective:**

The Chronic Care Model (CCM) has achieved widespread acceptance and reflects the core elements of patient-centred care in chronic diseases such as cardiovascular diseases (CVD). In the Netherlands the extent to which CVD patients receive care congruent with the CCM is unknown. The main objectives of this study were to validate the 20-item Patient Assessment of Chronic Illness Care (PACIC) and the 11-item (PACIC-S) in the Netherlands among CVD patients and investigate the validity, reliability, and sensitivity to change of both instruments.

**Methods:**

The Dutch version of the PACIC and PACIC-S were tested among 1484 CVD patients (out of 2760; response rate 54%) enrolled in Disease Management Programmes (DMPs) at T0 and 1167 respondents (out of 2545; response rate = 46%) at T1. Five hundred-eighty-five CVD patients filled in the questionnaire at both T0 and T1. We tested the instrument by means of structural equation modeling, and examined its construct validity, reliability and sensitivity to change. Reliability of the instrument was assessed by determining the statistical coherence of the scaled items. Internal consistency of the subscales was assessed by calculating Cronbach’s alphas and correlations between the PACIC and PACIC-S. We investigated the sensitivity to change of the original PACIC and the PACIC-S with paired t-tests among CVD patients in DMPs who filled in the questionnaire at both T0 and T1 (N = 585).

**Results:**

The confirmatory factor analyses revealed good indices of fit with the PACIC and PACIC-S. Internal consistency as represented by Cronbach’s alphas were also good. Correlations between the PACIC and PACIC-S subscales were excellent: 0.98 at both T0 and T1. Paired t-tests results show that the PACIC and PACIC-S improved significantly over time (*p* < 0.01).

**Conclusions:**

The psychometric properties of the Dutch PACIC and PACIC-S were satisfactory and it is sensitive to change, rendering it a valid and reliable instrument for assessing chronic illness care among CVD patients.

## Introduction

Chronic diseases such as cardiovascular diseases (CVD) are major causes of death and disability worldwide with rising prevalence [1]. They pose a significant health threat and an increasing challenge to health care systems [2]. Despite advances in treatment, patients with chronic diseases do not always receive optimal care [3-10]. Current care is often event-driven, despite evidence that a structured, proactive approach helps reduce the burden of several chronic diseases [11]. Because the causes of chronic diseases, such as CVD are complex, treatment should be multifaceted, integrated, and tailored to patient needs.

Disease management programmes (DMPs) aim to improve the efficiency and effectiveness of chronic care delivery [10] by combining patient-related, professionally-directed and organisational interventions [12,13]. In the Netherlands, DMPs are often based on the Chronic Care Model (CCM) [14-17]. The idea is to transition chronic care from acute and reactive to proactive, planned, and population-based [5]. A recent literature review reaffirms the notion that redesigning care using the CCM leads to improved patient care and better health outcomes [13]. The model provides an organised multidisciplinary approach to care for patients with chronic diseases. Glasgow and colleagues [18] developed the “Patient Assessment of Chronic Illness care” (PACIC) to assess patients’ perspective of alignment of primary care to the CCM. The PACIC has been used both nationally and internationally as an instrument to evaluate the delivery of CCM activities for a variety of chronic health conditions including, diabetes, osteoarthritis, depression, asthma, hypertension and COPD [19-24]. The paradigm for high-quality chronic illness care now seeks to promote a fuller understanding of the patient’s preferences in order to improve self-management abilities, activate and/or empower patients [25,26]. No data are available to date showing the extent to which current primary care for the CVD patients is CCM-compliant.

In this article, we describe the psychometric testing of the PACIC and PACIC-S among CVD patients enrolled in DMPs participating in quality improvement projects focused on chronic care in the Netherlands. Our objectives are to validate the PACIC and PACIC-S among CVD patients in the Netherlands and test its validity, reliability, and sensitivity to change.

## Methods

### Study population

Our study included 1484 CVD patients (out of N = 2760; response rate =54%) enrolled in eight DMPs in various regions in the Netherlands at T0. These eight DMPs consisted of 38 primary care practices. This sample was further reduced to 1321 to eliminate respondents with missing responses on all PACIC items. About a year later a questionnaire (T1) was sent to all CVD patients participating within the DMPs. A total of 1167 respondents filled in the questionnaire (out of 2545; response rate = 46%). Five hundred-eighty-five CVD patients (about a third of our sample) filled in the questionnaire at both T0 and T1.

### Setting

The study is funded by a national programme on “disease management of chronic diseases” carried out by ZonMw (Netherlands Organisation for Health Research and Development) and commissioned by the Dutch Ministry of Health. The study was extended for the cardiovascular DMPs ‘Vitale Vaten’ and received additional support and funding from the Heart Foundation. The following eight cardiovascular DMPs were selected by ZonMw based on quality and relevancy criteria retrieved from their project proposals: Onze Lieve Vrouwe Gasthuis (OLVG), Stichting Eerstelijns Samenwerking Achterveld (SESA), Regionale Organisatie Huisartsen Amsterdam (ROHA), Stichting Gezondheidscentra Eindhoven (SGE), Gezondheidscentrum Maarssenbroek, Ziekenhuis Rijnstate, Universitair Medisch Centrum St Radboud, and Wijkgezondheidscentra Huizen. All eight DMPs focused on patients at risk of having (another) cardiovascular incident. The DMPs comprise a variety of collaborations (mostly general practitioners, physiotherapists, and dieticians) undergoing internal practice redesign to improve chronic care management in primary care practices. They address shortcomings in acute care models by identifying elements that encourage high-quality chronic disease care in the early stages of care for patients with CVD [27,28]. Each programme consists of a combination of patient-related (self-management interventions such as patient education on lifestyle, regulatory skills, and proactive coping), professionally directed (implementation of care standards, protocols supported by information and communications technology tools such as integrated information systems), and organisational interventions (new care provider collaborations, reallocation of tasks, more effective information transfer and appointment scheduling, case management, employing new types of health professionals, redefining professionals’ roles and redistributing their tasks). This implementation of a combination of patient-related, professionally directed and organisational interventions led to improved integrated chronic care delivery as assessed by professionals [17].

The professionals personally handed the questionnaire to patients at consultations or mailed it to patients’ homes. All non-respondents received a reminder and another copy of the questionnaire a few weeks later. The study was approved by the ethics committee of the Erasmus University Medical Centre of Rotterdam in September 2009. Data were collected anonymously and treated confidentially to protect sensitive patient information.

### Measures

Patients assessed chronic illness care (PACIC) with a 20-item questionnaire comprising five pre-defined subscales: patient activation (3 questions), delivery-system/practice design (3), goal setting/tailoring (5), problem solving/contextual (4), and follow-up/coordination (5). The five-point response scale ranged from ‘almost never’ to ‘almost always’ with higher scores indicating a more frequent presence of the respective aspect of chronic care. The PACIC score was the sum of participants’ responses divided by 20. Scores thus ranged from 1 to 5 with higher scores indicating a greater perception of involvement in self-management and receipt of chronic care delivery [18]. In addition, we investigated the 11-item PACIC-S questionnaire [21]. While Gugiu and colleagues [21] used a modified version of the original PACIC for their study (they employed an 11-point percentage scaling from 0%-100%), we used scaling of the original PACIC.

Reliability of the instrument was assessed by determining the statistical coherence of the scaled items, which reflects the degree to which they measure the intended aspect of chronic care. Validity is the degree to which a scale measures what it is intended to measure; here we focused on the construct validity of the questionnaire and sensitivity to change.

### Analysis

Our analyses involved the following seven steps.

1. The sample characteristics were analysed using descriptive statistics.

2. We data-screened the items by examining the number of missing and the mean and standard deviation of each item.

3. To verify the factor structure of the 20-item and 11-item questionnaires we executed confirmatory factor analysis using the LISREL programme [29]. Listwise deletion of cases with missing data resulted in N = 1158 at T0.

4. To test the measurement models, we used indices of model fit whose cut-off criteria were proposed by Hu and Bentler [30]. First, the overall test of goodness-of-fit assessed the discrepancy between the model implied and the sample covariance matrix by means of a normal-theory weighted least-squares test. A plausible model has low, preferably non-significant *χ*^2^ values. However, Chi-square is overly sensitive in a large sample (over 200), leading to difficulty in obtaining the desired non-significant level [31]. Second, we used the Standardized Root Means square Residual (SRMR), which is a scale-invariant index for global fit ranging between 0 and 1. SRMR values below 0.08 indicate a good fit. Third, we calculated the Incremental Fit Index (IFI), which compares the independent model (i.e., observed variables are unrelated) to the estimated model. IFI values are preferably larger than 0.95.

5. The Dutch PACIC and PACIC-S was also tested on an imputed dataset by replacing missing values with the mean resulting in N = 1321.

6. Internal consistency of the subscales was assessed by calculating Cronbach’s alphas and correlations between the PACIC and PACIC-S.

7. We investigated the sensitivity to change of the original PACIC and the PACIC-S among CVD patients who filled in the questionnaire at both T0 and T1 (N = 585) to assess its ability to accurately detect changes. Paired t-tests were used to evaluate the sensitivity of the PACIC and PACIC-S to detect system improvements for CVD patients enrolled in DMPs.

## Results

### Sample characteristics

Table 1 displays characteristics of the study sample at T0. Of the 1321 respondents, 47% were female, 37% had a lower educational level, and 74% were married. Mean age was 63.77 ± 10.18 years (range: 29–91 years). We also assessed comorbidity among our study population. The majority of the respondents (61%) reported having at least one other chronic disease such as osteoarthritis (24%), severe spine conditions (17%), lung diseases (10%), diabetes (8%), or stroke (7%). The mean overall PACIC score of CVD patients measured with the 20-item instrument at T0 was 2.68 ± 0.86 and with the 11-item PACIC-S; 2.63 ± 0.86.

**Table 1:** Descriptive statistics

	**CVD patients ** **N = 1.321**
Mean age (years)	63.77 ± 10.18 (29–91)
Female subjects	47%
Married/living in partnership	74%
Low educational level	37%
Comorbidity	61%
Mean score on the 20 item PACIC	2.68 ± 0.86 (1–5)
Mean score on the 11 item PACIC-S	2.63 ± 0.86 (1–5)

Data are expressed as means ± standard deviation or *n* (%). CVD = cardiovascular disease.

### Datascreening

Table 2 shows the mean, standard deviation and the number of missing responses on each PACIC item. These results indicate a relatively high score on item 5 ‘Satisfied that my care was well organized’. All items had less than 10% missing responses.

**Table 2:** Item characteristics and factor loadings of the PACIC and PACIC-S (N = 1321)

**Item**		**Missing**	**Mean**	**Sd**
1. Asked for my ideas when made a treatment plan	1216	105 (7.9%)	2.96	1.30
**2. Given choices on treatment to think about**	1201	120 (9.1%)	2.82	1.31
3. Asked to talk about any problems with my medicines or their effects	1214	107 (8.1%)	2.92	1.41
4. Given a written list of things I should do to improve my health	1206	115 (8.7%)	2.81	1.39
**5. Satisfied that my care was well organized**	1216	105 (7.9%)	4.06	0.97
6. Shown how what I did to take care of my illness influenced my condition	1210	111 (8.4%)	3.37	1.31
7. Asked to talk about my goals in caring for my illness	1198	123 (9.3%)	2.66	1.31
**8. Helped to set specific goals to improve my eating or exercise**	1203	118 (8.9%)	2.83	1.36
**9. Given a copy of my treatment plan**	1208	113 (8.5%)	1.88	1.15
**10. Encouraged to go to a specific group/class to help me cope with my chronic illness**	1192	129 (9.7%)	1.93	1.16
**11. Asked questions, either directly or on a survey, about my health habits**	1210	111 (8.4%)	3.71	1.30
12. Sure that my doctor or nurse thought about my values and my traditions when they recommended treatment to me	1202	119 (9.0%)	3.44	1.29
**13. Helped to make a treatment plan that I could do in my daily life**	1201	120 (9.0%)	2.52	1.39
**14. Helped to plan ahead so I could take care of my illness even in hard times**	1195	126 (9.5%)	2.26	1.28
**15. Asked how my chronic illness affects my life**	1192	129 (9.7%)	2.42	1.35
**16. Contacted after a visit to see how things were going**	1197	124 (9.4%)	2.06	1.25
17. Encouraged to attend programmes in the community that could help me	1198	123 (9.3%)	1.93	1.16
18. Referred to a dietician, health educator, or counselor	1198	123 (9.3%)	2.56	1.44
**19. Told how my visits with other types of doctors, like the eye doctor or surgeon, helped my treatment**	1193	128 (9.7%)	2.52	1.45
20. Asked how my visits with other doctors were going	1195	126 (9.5%)	2.08	1.25

^*^Items in bold are the 11-item PACIC-S.

### Confirmatory factor analysis with 20 items

Indices of model fit showed sufficiency (Table 3). The significant Normal Theory Weighted Least Square *χ*^2^ statistic was 2200.005. IFI was above cut-off value of 0.95 and SRMR was below the cut-off value of 0.08. All indices indicated that the model was acceptable [30]. The model on imputed data resulted in comparable factor loadings and its model indices showed good fit.

**Table 3:** Model fit of the full and short models

	***Χ***^**2**^**(p)**	**IFI**	**SRMR**
Model 1: 20 item PACIC (N = 1158)	2200.005 (0.00)	0.983	0.0611
Model 2: 11 item PACIC-S (N = 1158)	710.641 (0.00)	0.980	0.0497
Model 3: 20 item PACIC on imputed data (N = 1321)	2408.259 (0.00)	0.971	0.0620
Model 4: 11 item PACIC-S on imputed data (N = 1321)	766.445 (0.00)	0.964	0.0494

### Confirmatory factor analysis with 11 items

Indices of model fit showed sufficiency (Table 3). The significant Normal Theory Weighted Least Square *χ*^2^ statistic was 710.641. IFI of the PACIC-S was above cut-off value of 0.95 and SRMR was far below the cut-off value of 0.08. The model on imputed data resulted in comparable factor loadings and its model indices also showed good fit.

### Internal consistency and inter-correlations

We investigated internal consistency with Cronbach’s alpha. Cronbach’s alpha ranged from good (PACIC-S of 0.88 at both T0 and T1) to excellent (PACIC of 0.93 at T0 and 0.94 at T1). The correlations between the 20-item PACIC instrument and the 11-item PACIC-S were excellent; 0.98 at both T0 and T1.

### Sensitivity to change

We investigated the sensitivity to change of the PACIC and PACIC-S to assess its ability to accurately detect changes if they occurred. Five hundred-eighty-five CVD patients filled in the questionnaire at both T0 and T1. Both instruments were responsive to system improvements. Paired t-tests results showed that the PACIC scores improved significantly at *p* < 0.001 (Table 4). We also tested the sensitivity to change of the PACIC-S. Paired t-tests results also showed that the scores improved significantly (*p* < 0.001) (Table 4).

**Table 4:** Sensitivity to change of the PACIC and PACIC-S (N = 585)

	**Baseline assessment**		**Follow-up assessment**		**Change scores (T1-T0)**		**Significance of difference**^**a**^
	**M**	**SD**	**M**	**SD**	**M**	**SD**	***P*-value**
20 item PACIC	2.71	(0.84)	2.81	(0.82)	0.11	(0.77)	< 0.001
11 item PACIC-S	2.66	(0.84)	2.77	(0.82)	0.12	(0.80)	< 0.001

^a^ Significance of difference between scores at baseline and follow-up. Paired t-tests were used to test significance of difference.

### Alignment of primary care to the CCM

Table 5 displays the average PACIC scores of Dutch CVD patients in comparison to baseline PACIC scores tested by Glasgow and colleagues [14] in the Unites States, diabetes patients in the US [19], German osteoarthritis patients [20], COPD patients in the Netherlands [32] and diabetes patients in the Netherlands [24].

**Table 5:** Average PACIC scores comparison between the CVD patients in the Netherlands, PACIC scores tested in the Unites States; Diabetes patients in the US; German osteoarthritis patients; COPD patients in the Netherlands and diabetes patients in the Netherlands

	**20-item PACIC scores**		
**Samples**	**M**	**SD**	**N**
Overall baseline scores Glasgow (patients with hypertension, arthritis, depression, diabetes and asthma) in the US	2.6	(1.0)	266
Diabetes patients in the US	3.2	(0.9)	641
German osteoarthritis patients	2.4	(1.1)	236
Dutch diabetes patients	3.2	(1.0)	88
Dutch COPD patients	2.9	(0.9)	917
Dutch CVD patients in the current sample	2.7	(0.9)	1321

## Discussion

This study aimed to validate the PACIC and PACIC-S in the Netherlands as an instrument to assess CVD patients’ perspectives of alignment of primary care to the CCM. In addition, we aimed to evaluate improvements made by DMPs as assessed by CVD patients enrolled in Dutch DMPs. The confirmatory factor analysis, internal consistency, inter-correlations and sensitivity to change analyses with both the 20-item PACIC and 11-item PACIC-S showed that the psychometric properties of the instruments are satisfactory. Both instruments revealed good indices of fit as indicated by the high reliability coefficients, showing good internal consistency. Furthermore, both the PACIC and PACIC-S consistently showed their ability to detect improvements as assessed by CVD patients in the delivery of chronic illness care.

In case the original PACIC is considered too lengthy, the PACIC-S is a good alternative to assess if primary care for CVD patients is CCM-compliant.

The mean scores on the PACIC among CVD patients in the Netherlands were similar to the baseline scores found by Glasgow and colleagues in the US [18] among patients with a variety of chronic conditions. The mean PACIC scores of CVD patients were lower than COPD patients in the Netherlands [32], lower compared to patients with diabetes in both the Netherlands [24] and the US [19], but higher compared to the scores of osteoarthritis patients in Germany [20]. These results suggest that primary care for CVD patients – as perceived by patients – is more structured than for patients with osteoarthritis. The relatively higher PACIC scores for diabetes and COPD patients may be explained by earlier attention for enhancing structured care [20].

It is important to note that our study involves several limitations. Retest reliability, for example, was not examined. However, it has been debated that test-retest reliability may be less useful than internal consistency reliability [33]. While Spicer and colleagues [34] recognize the PACIC as a formative rather than a reflective measure, which makes traditional analyses of its factorial validity (and internal consistency) inappropriate, our findings suggest the PACIC to be a reflective measure. Furthermore, we did not investigate if improved PACIC or PACIC-S scores actually led to improved patient outcomes. Further research is necessary to show if the PACIC is not only useful as an assessment tool, but can also be used as a decision-making tool, showing which elements of chronic care delivery need further improvements leading to improved patient outcomes. We also did not have an objective measure that chronic care delivery was indeed improved even though the programmes were implemented with the intent to improve chronic care delivery. Finally, we investigated the PACIC among CVD patients enrolled in DMPs only. These practices redesigned their healthcare delivery addressing shortcomings in acute care models by identifying elements that encourage high-quality chronic disease care in the early stages of care for patients with CVD. While Spicer and colleagues [34] concluded that sensitivity to change of the PACIC has not been reported to date, this is the first study showing that both the PACIC and PACIC-S are sensitive to changes in primary healthcare delivery. After implementation of a combination of patient-related, professionally directed and organisational interventions to improve chronic care delivery both the PACIC and PACIC-S scores improved significantly.

We conclude that the psychometric properties of the PACIC and the PACIC-S among CVD patients are good and that both instruments are promising to assess CVD patients’ perspective of alignment of primary care to the CCM. The 11-item PACIC-S is a less burdensome instrument compared with the 20-item PACIC to measure patient assessment of chronic care delivery. Furthermore, the generic nature of the PACIC items makes it possible to assess patients’ perspective on chronic care delivery also if they have more than one chronic condition. In addition, the PACIC and the PACIC-S are promising to evaluate the level and nature of improvements made in DMPs as proven by their sensitive to change.

## Competing interests

The authors declare that they have no competing interests.

## Authors’ contribution

AN drafted the design for data gathering. JC and AN were involved in acquisition of subjects and data, performed statistical analysis and interpretation of data. JC drafted the manuscript and AN helped drafting the manuscript and contributed to refinement. Both authors have read and approved its final version.
